# Doctors' Wills

**Published:** 1914-04-04

**Authors:** 


### Doctors' Wills.

Mr. G. B. Rosewall, M.R.C.S., L.S.A., aged ninety-four, formerly medical officer of health for the West Cornwall District, has left estate of the gross value of £29,520.

Dr. Matthew Martin, M.B., C.M., J.P., of Glencairn, Parkhead, Glasgow, for thirty years a member of the Glasgow Parish Council, left estate of the value of £3,858.

Dr. Albert Charles Lewis Günther, Ph.D., M.D., LL.D., F.R.S., F.L.S., F.Z.S., formerly Keeper of the Zoological Department of the British Museum, left estate, of which £2,362 is net personalty.

Dr. Julia Anne Hornblower Cock, M.D. (Brux.), L.R.C.P.I., L.M., L.R.C.S., of Nottingham Place, W., medical examiner under the Board of Education, Dean of the London (Royal Free Hospital) School of Medicine for Women, and senior physician to the new Hospital for Women, has left estate of which £956 is net personalty.

Mr. Henry Albert Reeves, the orthopædic surgeon, has died at the age of seventy-three. Educated at Middlesex Hospital, where he held house appointments, he became demonstrator of anatomy at the London Hospital, and served on the staffs of the Central London Ophthalmic Hospital and the East London Children's Hospital. During the Franco-German War he was surgeon to the British Society for Aid to the Sick and Wounded, and afterwards became senior surgeon to the Hospital for Women, Soho.

